# Volume XXI

**Published:** 1881-08

**Authors:** 


### VOLUME XXI.

With this number commences another year of the JOURNAL, and our subscribers will find inclosed their bills for the ensuing year. This begins the third year of the present management. The price of the JOURNAL during this time has been, and for the present will continue to be, two dollars, one-third less than at any previous time. It will readily be seen that at this price there can be but one way to make ends meet—to see that all dues are promptly collected. This being the case, we feel free to call the attention of our friends to the necessity for early remittances. During the past year we have on the whole had no reason to complain; while a few have been somewhat dilatory or have perhaps entirely neglected to respond, of the large majority we have only the request to make that they do as well in the future.

